# Early spontaneous cessation of subdural drainage after burr hole evacuation of chronic subdural hematoma and risk of recurrence

**DOI:** 10.1371/journal.pone.0285750

**Published:** 2023-05-17

**Authors:** Mads Hjortdal Grønhøj, Thorbjørn Søren Rønn Jensen, Bjarni Johannsson, Kåre Fugleholm, Frantz Rom-Poulsen

**Affiliations:** 1 Department of Neurosurgery, Odense University Hospital, Odense, Denmark; 2 Department of Clinical Research and BRIDGE—Brain Research Inter-Disciplinary Guided Excellence, University of Southern Denmark, Odense, Denmark; 3 Department of Neurosurgery, Rigshospitalet, Copenhagen, Denmark; Duke University Medical Center: Duke University Hospital, UNITED STATES

## Abstract

**Objective:**

Subdural drainage reduces recurrence after evacuation of chronic subdural hematoma (CSDH). In the present study, the authors investigated the dynamics of drain production and potentially contributing factors for recurrence.

**Method:**

Patients treated with a single burr hole evacuation of CSDH between April 2019 and July 2020 were included. Patients were also participants in a randomized controlled trial. All patients included, had a passive subdural drain for exactly 24 hours. Drain production, Glasgow Coma Scale score, and degree of mobilization was recorded every hour for 24 hours. A CSDH successfully drained for 24 hours is referred to as a “case”. Patients were followed for 90 days. Primary outcome was symptomatic recurrent CSDH requiring surgery.

**Results:**

A total of 118 cases from 99 patients were included in the study. Of the 118 cases, 34 (29%) had spontaneous drain cessation within the first 0–8 hours after surgery (Group A), 32 (27%) within 9–16 hours (Group B), and 52 (44%) within 17–24 hours (Group C). Hours of production (P < 0.000) and total drain volume (P = 0.001) were significantly different between groups. The recurrence rate was 26.5% in group A, 15.6% in group B, and 9.6% in group C (P = 0.037). Multivariable logistic regression analysis show that cases in group C (OR: 0.13, P = 0.005) are significantly less likely to recur compared to group A. Only in 8 of the 118 cases (6.8%), the drain started draining again after an interval of three consecutive hours.

**Conclusions:**

Early spontaneous cessation of subdural drain production seems to be associated with increased risk of recurrent hematoma. Patients with early cessation of drainage did not benefit from further drain time. Observations of the present study indicate personalized drainage discontinuation strategy as a potentially alternative to a specific discontinuation time for all CSDH patients.

## Introduction

Chronic subdural hematoma (CSDH) is a common neurosurgical condition that mainly affects people aged over 70–75 years [1, 2]. A major challenge in CSDH treatment is the risk of recurrence causing repeated surgery, with prolonged hospital stays and higher morbidity and mortality [3–7]. With an aging population, evacuation of CSDH is expected to become the most frequent cranial procedure [5, 8]. This calls for optimized treatment to reduce the recurrence risk.

Surgical techniques for CSDH vary from country to country, but evidence supports the beneficial effect of postoperative subdural drainage on recurrence and mortality [6, 9–11]. Different drainage techniques have been investigated (subgaleal, subdural, or continuous irrigation and drainage), but consensus conclusions are lacking [9, 12–14]. Furthermore, the optimal duration of subdural drainage is not clarified.

We believe that current practice in most treatment centers is drain removal after a specific postoperative period (e.g. 24 hours or 48 hours). Insight into drain mechanisms—including time-dependent drain production—could help the development of personalized drainage durations.

### Objectives

*First*, what are the dynamics of drainage production? When does the draining start and stop, and is this important for the total volume of CSDH fluid drained? *Second*, are the duration of drainage (the total hours of ‘active drain’) and the final drained volume of CSDH fluid important for recurrence? *Third*, is ‘active drainage’ dependent on patient mobilization and changes in postoperative Glasgow Coma Scale score (GCS)? *Fourth*, if the drain has not drained for a certain period of time, will it then start draining again or can it be removed?

## Methods

### Ethical approval

The study was approved by the Danish Data Protection Agency (journal no.18/20513) and the Scientific Ethical Committee of Copenhagen (project ID: S-20180010). Oral and written informed consent was obtained from the participants, whereby they accepted participation in the study and gave permission to data extraction from the electronical medical record. All data was registered in an online database (REDCap—Research Electronic Data Capture system) via an encrypted connection and anonymized to fulfil the demands for data protection.

This study was designed and conducted by members of the Danish Chronic Subdural Hematoma Study (DACSUHS), a national steering group with representatives from all four neurosurgical departments in Denmark. DACSUHS coordinates CSDH management and research in Denmark.

### Patients and setting

Mentally competent patients were included from two institutions (Odense University Hospital and Rigshospitalet) between April 2019 and July 2020. A subdural hematoma successfully drained for 24 hours is referred to as a “case”, meaning that a patient with a unilaterally drained CSDH represents one case whereas a patient with a bilaterally drained CSDH represents two cases.

Recurrence in patients with bilateral hematomas is linked to the symptomatic side (case) that required repeated surgery (Table 1).

**Table 1:** Patients with recurrent bilateral hematomas.

Patients	Case	Drainage group	Symptomatic recurrence	Repeated surgery
No 1	Right	A	Yes	Yes
	Left	A	No	No
No 2	Right	B	No	No
	Left	A	Yes	Yes
No 3	Right	A	Yes	Yes
	Left	A	Yes	Yes
No 4	Right	B	Yes	Yes
	Left	B	Yes	Yes

No = Number

**Fig 1:**
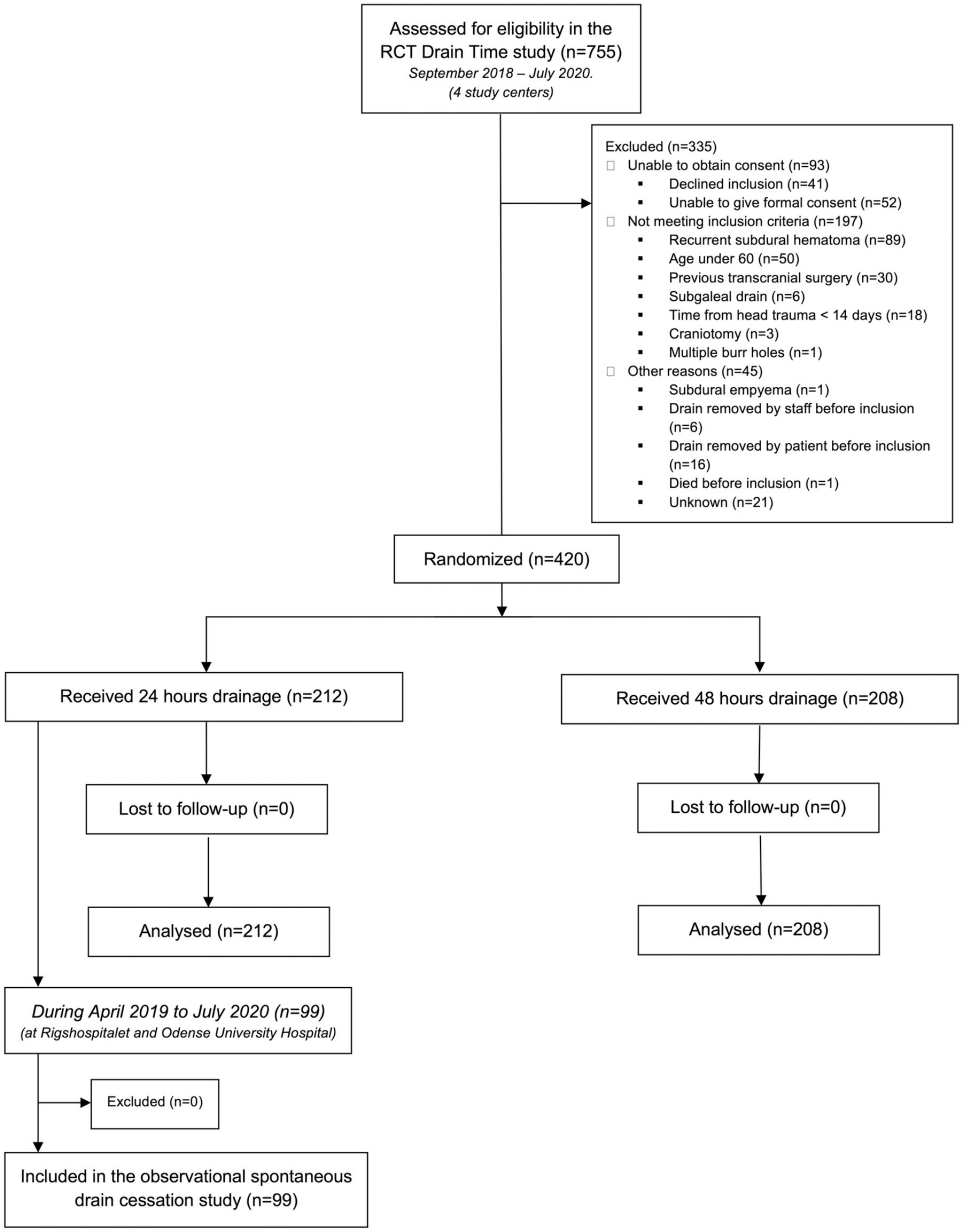
Included patients. Fig 1 is a flowchart showing patients included in the randomized controlled trial (Drain Time) and patients included in the present substudy. RCT (randomized controlled trial).

The patients included in the present study were also participants in the nationwide randomized controlled trial of drainage time, the “Drain-Time” study (running from September 2018 to July 2020) [15], which included patients from all four neurosurgical departments in Denmark (Fig 1). Patients included in the present study were those who had their drains removed after 24 hours. No drains were removed before 24 hours. Patients aged 60 years or above who had single burr hole evacuation of CSDH were eligible for inclusion. Hematoma subtypes were defined based on the preoperative CT scan as previously described [16]. Exclusion criteria were previous intracranial surgery, known head injury in the 14 days prior to surgery, multiple burr holes, and subgaleal drain placement [15].

### Surgery, follow-up, and outcome

The surgical management of symptomatic CSDH has been described in a previous publication [15]. Patients were followed for 90 days. The primary outcome was symptomatic recurrent CSDH requiring surgery. A control CT scan was only indicated if the original symptoms either did not improve or recurred, or if new symptoms developed.

### Drain monitoring and clinical assessment

All included patients received passive subdural drainage and were allowed immediate postoperative mobilization with the drain open and attached by an adhesive bandage at the level of the clavicula. Recovery nursing staff recorded drain production, Glasgow Coma Scale (GCS) score, and degree of mobilization (in bed, sitting in chair, or walking around) every hour for 24 hours. The drainage bag was regularly emptied, and the volume was read in a measuring flask. The accuracy of the drained volume was +/- 5 milliliters (ml). The draining tube (from brain to drainage bag) can contain a total of approximately 1.5–2 ml.

### Statistics

The patients were retrospectively divided into three groups (timewise identical periods—first 8 hours, middle 8 hours, last 8 hours) depending on the time of spontaneous cessation of drainage: Group A (drainage stopped spontaneously within 0–8 hours post-surgery), Group B (drainage stopped spontaneously within 9–16 hours post-surgery), and Group C (drainage stopped spontaneously within 17–24 hours post-surgery).

Clinical and demographic characteristics were assessed for normality by Shapiro-Wilk test followed by Kruskal-Wallis one-way analysis of variance to assess equivalence between the respective variables in the three groups (Table 2).

**Table 2:** Clinical and demographic characteristics.

**Patients**	**Drainage 0–8 h (N = 29)**	**Drainage 9–16 h (N = 27)**	**Drainage 17–24 h (N = 43)**	**P-value**
Sex (male)	23 (79.3%)	24 (88.9%)	30 (69.8%)	0.231
Age	75 (62–81)	79 (73–83)	75 (72–82)	0.057
Known head trauma	22 (75.9%)	15 (55.6%)	25 (58.1%)	0.176
GCS pre-operatively	15 (14–15)	15 (14–15)	15 (14–15)	0.954
Anticoagulant therapy	7 (24.1%)	5 (18.5%)	13 (30.2%)	0.469
Antiplatelet therapy	10 (34.5%)	9 (33.3%)	11 (25.6%)	0.392
Duration of surgery (min)*	34 (29–53)	45 (32–53)	40 (30–55)	0.181
**Haematomas (cases)**	**Drainage 0–8 h (N = 34)**	**Drainage 9–16 h (N = 32)**	**Drainage 17–24 h (N = 52, base)**	
Location (right sided)	18 (52.9%)	15 (46.9%)	30 (57.7%)	0.567
Hematoma type				
*Homogeneous*	14 (41.2%)	13 (40.6%)	23 (45.1%)	0.530
*Membranous*	14 (41.2%)	10 (31.3%)	16 (31.4%)	
*Mixed*	3 (8.9%)	8 (25.0%)	10 (19.6%)	
*Sedimented*	3 (8.9%)	1 (3.1%)	2 (3.9%)	
Haematoma size	88 (57–119)	113 (88–155)	103 (79–139)	0.482
Midline shift*	6 (2–9)	6 (3–9)	7 (4–10)	0.611
Production (ml)	30 (10–40)	50 (35–70)	85 (40–165)	**0.001**
Hours of production	2 (1–3)	11 (8–13)	20 (16–23)	**<0.000**
Drainage start (hours after insertion)	0 (0–0)	0 (0–1)	0 (0–2)	0.728
Recurrence	9 (27.3%)	5 (15.6%)	5 (9.6%)	**0.037**
Death	0 (0%)	2 (6.3%)	2 (3.9%)	0.459

Data are median (InterQuatile Range, IQR) or number (%) followed by their respective p-values obtained by ANOVA analysis. ml (millilitres), min (minutes).

*Some data missing

Two multivariable logistic regression models were used to investigate the effects of independent variables on CSDH recurrence in the three groups. The dependent variable for the first model was recurrence of CSDH as a binary variable regardless of timeframe. Independent variables for the model were age, gender, drain-cessation group as a categorical variable (A, B, and C), size of hematoma (converted to centiliters for easier interpretation of coefficients), type of hematoma (unilateral or bilateral), use of any type of anticoagulants, pre-operative GCS and midline shift.

The second multivariable logistic regression model also had recurrence of CSDH as the dependent variable. Independent variables for this model were age, gender, hours of drain production, size of hematoma, type of hematoma (unilateral vs. bilateral), use of anticoagulants, pre-operative GCS and midline shift. This model was employed to independently analyze hours of production as a covariate; this could not be estimated correctly in the first model due to confounding with drain-cessation groups.

In addition, a third multivariable logistic regression model examined the relationship between recurrence and volume drained with the same covariates.

Significant findings were reported as odds ratios with a discrepancy of α = 0.05 and no adjustment to protect the results of this explorative study from type II errors.

Data analyses were performed using Stata 16. (StataCorp. 2018. Stata Statistical Software: Release 16. College Station, TX: StataCorp LLC).

## Results

A total of 118 cases from 99 patients were included in the study. Their demographic and clinical characteristics are shown in Table 2. No patients were lost to follow-up. Groups were well matched regarding baseline demographic and clinical characteristics. Haematoma characteristics were also equally distributed in the three groups, including no significant differences in haematoma subtype, haematoma size, or midline shift.

Nineteen patients had bilateral hematomas, and all were operated on both sides. Four patients experienced symptomatic recurrence within three months and were reoperated (Table 1). Two patients were reoperated on both sides, and two patients on one side only (case recurrence rate of 16%).

### Production dynamics–univariate analysis of draining parameters

Of the 118 cases, 34 (29%) had spontaneous drain cessation within the first 0–8 hours after surgery (Group A), 32 (27%) had spontaneous drain cessation 9–16 hours after surgery (Group B), and 52 (44%) had spontaneous drain cessation 17–24 hours after surgery (Group C) (Table 2).

**Fig 2:**
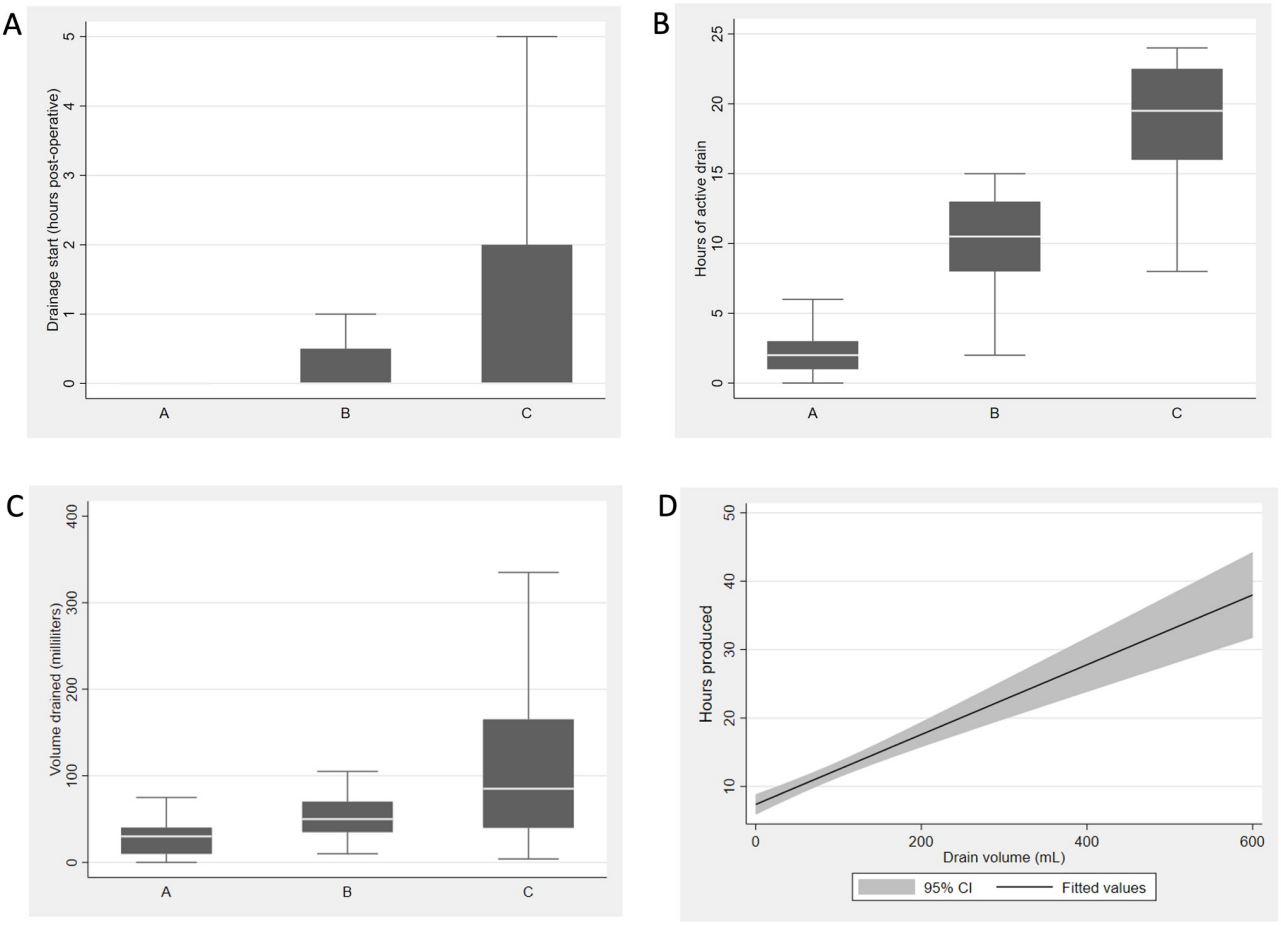
Drain production dynamics. A shows the median drain start time in each of the three groups. Data are presented as means and standard deviation. The median drain start time was 0 hours post-surgery for every group. InterQuartiel Range (IQR) was 0–0.5 for group B and 0–2 for group C. B shows the median drainage time (hours of/with production). The median drainage time was 2 hours (IQR 1–3) in group A, 10.5 hours (IQR 8–13) in group B and 19.5 hours (IQR 16–22.5) in group C. C shows the total drain production (volume, mL). Group A; 30 mL (IQR 10–40), Group B; 50 mL (IQR 35–70) and Group C; 85 mL (IQR 40–165). D represents a univariable regressions analysis between total drain production (volume, mL) and total hours of production (P < 0.000, slope 0.051, 95% CI: 0.04–0.06).

A total of 72.9% of the drains began production immediately after surgery, of which 82.4% were in group A, 75% in group B, and 65.4% in group C. There was no significant difference between the groups in the median drain start time (Fig 2A).

However, hours of production were significantly different between groups (P < 0.000) (Fig 1B), as was the total drain volume (P = 0.001) (Fig 2C). There was a strong correlation between total drain production (volume) and total hours of production (P < 0.000) (Fig 2D).

The recurrence rate was 26.5% in group A, 15.6% in group B, and 9.6% in group C, which was significantly different (P = 0.037).

### Production dynamics and recurrence–multivariable logistic regression

The first multivariable logistic regression model was used to investigate the effect of age, gender, hematoma size, drain cessation group, bilateral hematomas, anticoagulant use and midline shift on recurrence. Compared to group A, cases in group C (OR: 0.13, P = 0.005) were significantly less likely to recur (Table 3). Compared to group A, cases in group B (OR: 0.24, P = 0.055) almost reached the level of significance, with a trend towards reduced risk of recurrence.

**Table 3:** Multiple logistic regression results.

Recurrence	OR	95% CI	P-value
Age	0.99	0.94–1.06	0.982
Gender	0.74	0.17–3.34	0.703
Drain class			
*<8 hours*	Base		
*8–15 hours*	0.24	0.06–1.03	*0*.*055*
*>16 hours*	0.13	0.03–0.54	**0.005**
Size of hematoma	1.18	1.03–1.35	**0.012**
Bilateral hematoma	1.70	0.38–7.61	0.485
Use of anticoagulants	0.53	0.14–2.01	0.348
Midline shift	1.06	0.93–1.22	0.364
Pre-operative GCS	0.31	0.07–1.34	0.116

Results of multiple logistic regression for predicting recurrence. OR = Odds ratio, 95% CI = 95% confidence interval.

Likewise, the size of hematoma (OR: 1.18) was correlated with recurrence, with a 17.7% increase in odds for every 100 mL of hematoma size.

The second multivariable logistic regression model was used to analyze the relationship between recurrence and total hours of production regardless of drain cessation time point and adjusted for age, gender, size of hematoma, bilateral occurrence, anticoagulant use, pre-operative GCS and midline shift (Table 4). We observed that a shorter duration of production was significantly correlated with increased risk of recurrence (OR 0.91, P = 0.024).

**Table 4:** Multiple logistic regression results for hours produced.

Recurrence	OR	95% CI	P-value
Age	0.99	0.93–1.05	0.760
Gender	0.90	0.20–4.02	0.897
Hours produced	0.91	0.85–0.99	**0.024**
Size of hematoma	1.16	1.02–1.31	**0.017**
Bilateral hematoma	1.81	0.41–7.93	0.433
Use of anticoagulants	0.59	0.16–2.18	0.430
Midline shift	1.07	0.94–1.23	0.285
Pre-operative GCS	0.32	0.07–1.35	0.122

Results of multiple logistic regression for predicting recurrence. OR = Odds ratio, 95% CI = 95% confidence interval

As bilateral hematomas did not increase the risk of recurrence (Table 4, P = 0.433), we did not explore further the difference between unilateral and bilateral hematomas.

The third multivariable logistic regression model adjusted for the parameters listed in Table 4, showed that the total volume produced is statistically important for the risk of recurrence (OR 0.98, 95% CI: 0.97–1.00, P = 0.044).

### Drainage in relation to mobilization and postoperative GCS score

No patients deteriorated within the first 24 hours post-surgery, and no patients had a fluctuating GCS score from hour to hour. Therefore, we could not investigate the importance of postoperative changes in GCS score in relation to drain production.

The distribution of production hours during mobilization (sitting up in bed, in chair, or walking around) and bed rest is shown in Table 5.

**Table 5:** Drainage during mobilization and bed rest.

Groups	Total hours of drainage	Total hours mobilized	Total hours of bed rest	Hours of drainage during mobilization / out of total hours mobilized	Hours of drainage during bed rest / out of total hours of bed rest
A: 0–8 (n = 29)	129	181	515	23 (18%) / 13%	106 (82%) / 21%
B: 9–16 (n = 27)	393	178	470	102 (26%) / 57%	291 (74%) / 62%
C: 17–24 (n = 43)	833	312	720	252 (30%) / 80%	581 (70%) / 81%

We observed that the majority of “active drain hours” occurred during bed rest in all three groups, simply because the patients spent the most time lying in bed. However, the production ratio is identical between "mobilization" and "bed rest" in all three groups, indicating that the drains are equally active regardless of whether the patients are mobilized or in bed.

In Group A, drain production did not restart despite mobilization between 9–24 hours after surgery.

### Drain stop and restart

In 8 of the 118 cases (6.8%), the drain started draining again after an interval of three consecutive hours. These cases are shown in Table 6.

**Table 6:** Restart of draining after a pause of 3 consecutive hours.

Case No	Group	Drain pause (hours)	Volume drained after pause (mL)	Total volume drained (mL)
Case 1	B	4	10 (20%)	50
Case 2	B	10	20 (29%)	70
Case 3	C	4	30 (20%)	150
Case 4	C	8	35 (23%)	150
Case 5	C	15	15 (27%)	55
Case 6	C	10	30 (27%)	110
Case 7	C	12	15 (12%)	125
Case 8	C	10	15 (17%)	90

mL = mililiter, No = number

We defined “restart of draining” as a drained volume of minimum 5 ml after the pause of three consecutive hours to avoid misinterpretation or to count fluid within the drain tube that has been pushed into the draining bag e.g. because of air in the system. The volume drained after the pause in these 8 cases was 12–29% of the total volume drained, and the median drained volume after the pause was 17.5 ml. In group A, no drains started draining again after they had stopped.

## Discussion

This study examined subdural drain dynamics after burr hole evacuation of CSDH. We report that a shorter duration of production (active drain) was significantly correlated with an increased risk of recurrence at three months postoperative follow-up.

Cases with an active drain until 17–24 hours after surgery had a significantly lower risk of symptomatic recurrence (compared to active drain < 8 hours after surgery) when adjusted for age, gender, size of hematoma, bilateral occurrence, midline shift, and anticoagulant use. We also observed that total volume produced is statistically significant for the risk of recurrence, as lower production increases the risk within the first 3 months post-operatively.

We know from previously randomized controlled trials that subdural drainage is important for reducing the risk of CSDH recurrence [9, 11], but the optimal duration of drainage is not yet clarified. With this observational cohort study, we have shed light on a group of patients who will experience symptomatic recurrence regardless of how long they keep their drains. This patient subgroup is highly interesting as the placement of a subdural drain seems to make no difference to their prognosis. We have not been able to identify important characteristics for this group, and we do not know why the drains do not start draining and/or only function for a very short time. Maybe the answer is simple, e.g. it was due to an obstruction of the drain, and one could speculate if this problem could be eliminated by placing a subperiosteal drain with/without suction or performing drain irrigation (manually or automatically) if the production is unsatisfactorily low.

Due to the composition of membranous and mixed hematomas, their viscosity, and the “intercavity” connections, we assume that these hematomas are more difficult to drain and that the drain is more prone to clogging. However, in our study, the different hematoma types were equally distributed in all three groups, with no significant differences. Perhaps the answer is more complex in that malfunction of the drain also depends on e.g. the hematoma composition at protein level [17] and/or brain stiffness [18], which have not yet been investigated.

While previous trials [15, 19] are investigating if there is an optimal postoperative time point to remove the drain, we took an opposite approach in this study by observing when the drains spontaneously stop. We observed that when the drain stopped in group A, the production did not restart at any time in the first 24 hours after surgery despite mobilization. In groups B and C, there were 8 cases (9.5% of groups B+C, 6.7% of the total cases) where the drains started draining again after a pause of three consecutive hours, and the volume drained after the pause was 12–29% (10–35 ml) of the total volume drained.

We report equal production ratios between "mobilization" and "bed rest" in all three groups. So, it seems to be subordinated to whether the patients are lying in bed or is mobilized, in relation to whether the drain is active or not.

The observations from this study indicate, that an individual drainage discontinuation strategy up to a maximum of 24 hours after surgery, could be a reasonable alternative to a specific discontinuation time for all patients. However, this question can only be answered through a randomized trial where a fixed drainage time is compared to an individual, production-dependent drainage time. One can argue for individual drainage times from both the health economic perspective and the patient perspective as some patients would be able to be discharged or transferred to another department/hospital earlier. The drain probably deters several patients from adequate mobilization (even though it is allowed), and here individual drainage time is likely to lead to earlier sufficient mobilization. From the nurse’s perspective, however, it is time-consuming to monitor drain production every hour. Therefore, the best overall solution in terms of the risk of CSDH recurrence is probably to ascertain the shortest possible—but equal—drainage time for all patients [20]. This should be combined with research on how to prevent early drain cessation.

### Study strengths and limitations

A strength of this study is that all patients received the national standard treatment and care for CSDH. The observation period was relatively short, and no patients were lost to follow up. The study design was simple, and the parameters of interest were easy to observe.

One of the limitations is the relatively low number of patients/cases. The patients participated in a randomized trial, why this observational substudy wasn’t designed nor powered for the analyses performed. This probably explains why we only observed a trend towards increased risk of recurrence in drain group B compared to group A. We were also not able to identify special characteristics for the cases in group A. Unfortunately, we did not examine the drains after they were removed, and this might have indicated whether they were obstructed or not.

Another limitation could be that we included patients with bilateral hematomas and registered each hematoma as a single case. It has been proposed that bilateral hematomas have different clinical and prognostic characteristics compared to unilateral haematomas. However, there were no demographic or prognostic differences between our groups, and only four patients (giving 6 cases) with bilateral hematomas, experienced symptomatic recurrence, thus justifying the approach of perceiving each hematoma as a single case.

## Conclusion

Early cessation of subdural drain production after single burr hole evacuation of CSDH seems to be associated with an increased risk of recurrent hematoma. Patients with early cessation of drainage did not benefit from further drain time. This group of patients is highly interesting, and a better understanding of the reasons for early drain cessation could significantly reduce the overall recurrence rate for CSDH. The findings of the present study, calls for a randomized trial to determine the potential beneficial effects of individual, personalized drainage time compared to a fixed drainage time in CSDH patients.

## Supporting information

S1 Table The study’s minimal underlying data set. This table contain the data set underlying the results described in the manuscript.
(XLS)
